# Experimental study on integrated desulfurization and denitrification of low-temperature flue gas by oxidation method

**DOI:** 10.1038/s41598-024-53765-y

**Published:** 2024-02-12

**Authors:** Yanyuan Bai, Yungang Wang, Haoran Xiu, Tao Liu, Li Zou, Guoqiang Liao, Qi Xiao

**Affiliations:** 1 Key Laboratory of Thermo-Fluid Science and Engineering (MOE), Xi’an Jiao Tong University, Xi’an, 710049 Shaanxi People’s Republic of China; 2 Science of Technology On Thermal Energy and Power Laboratory, Wuhan Second Ship Design and Research Institute, Wuhan, 4300764 Hubei People’s Republic of China

**Keywords:** Low-temperature flue gas, Oxidation method, Integration of desulfurization and denitrification, Fe-TiO_2_ catalysts, H_2_O_2_, Energy infrastructure, Environmental sciences

## Abstract

In this paper, TiO_2_ catalysts doped with different Fe contents (Fe-TiO_2_ catalysts) were prepared by coprecipitation method and the Fe loading capacity was optimized, and then the integrated pollutant removal experiment was conducted, in which TiO_2_ doped with Fe as catalyst and H_2_O_2_ as oxidant. The results show that under the condition of constant H_2_O_2_/(SO_2_ + NO) molar ratio, low concentration of SO_2_ can promote the oxidation and removal efficiency of NO, while high concentration of SO_2_ can inhibit the removal of NO_*x*_. The pollutant removal efficiency is proportional to the amount of catalyst, liquid–gas ratio and pH value of the absorbing solution. The optimal experimental conditions are H_2_O_2_/(SO_2_ + NO) molar ratio 1.5, space velocity ratio 10,000 h^−1^, H_2_O_2_ mass fraction 10 wt%, liquid gas ratio 10, pH 10. Correspondingly, NO oxidation efficiency reaches 88%, NO_*x*_ removal efficiency 85.6%, and SO_2_ is almost completely removed. The microstructure of the catalyst before and after the reaction was characterized, and the crystal structure did not change obviously. However, with the deepening of the reaction, the specific surface area of the catalyst decreases, and the catalytic effect decreases slightly.

## Introduction

In the context of the objectives of "carbon peak" and "carbon neutrality," the installed capacity of new energy generation, represented by solar and wind power, continues to grow^1^. Thermal power generation actively engages in facilitating deep load adjustments to accommodate the integration of new energy generation, thereby mitigating the inevitable impact of new energy integration on the power grid. However, when the units are low-load operation, the flue gas volume reduces, and the flue gas temperature reduces below the optimal catalytic temperature for Selective Catalytic Reduction (SCR), then the denitration efficiency is greatly reduced. Moreover, if the temperature remains below the low-temperature ammonia injection threshold for an extended period, ammonia can react with SO_3_ to produce ammonium hydrogen sulfate. This compound has a tendency to capture fly ash from the flue gas, leading to adhesion on heated surfaces and the catalyst, thereby causing blockages in surface voids, reduction in overall specific surface area, and a decrease in active catalytic sites. Consequently, the catalyst experiences diminished lifespan and decreased activity^2^.

During low-load operation, the flue gas flow rate decreases, resulting in reduced flow velocity as the flue gas passes through the pores of the catalyst. This scenario increases the risk of pore clogging due to ash accumulation within the catalyst pores^3^. Existing deep load adjustment units have implemented a series of strategies to mitigate the impact of low inlet flue gas temperatures on denitrification efficiency without altering the catalyst configuration. These methods include placing a portion of the reheater heating surface downstream of the SCR device to reduce upstream heat absorption and thereby elevate the inlet temperature to the SCR^4^. Additionally, a bypass arrangement in the water side of the reheater modifies the inlet water flow rate, effectively regulating the heat absorption by the flue gas^5^. However, most of the aforementioned measures are often implemented at the cost of sacrificing boiler thermal efficiency. Consequently, it becomes necessary to explore an alternative to traditional denitrification methods as a complementary solution for low-load operation of existing SCR systems. This novel approach should preserve the low-temperature catalytic activity of denitrification catalysts, effectively expanding the deep load adjustment capabilities of generating units, while simultaneously preventing the formation of adhesive ammonium hydrogen sulfate under low-temperature conditions, thereby safeguarding catalyst activity.

Hydrogen peroxide (H_2_O_2_), a cost-effective and highly oxidative green oxidant, presents itself as a candidate for this alternative approach. It achieves oxidation-based denitrification without inducing secondary pollution, effectively addressing both denitrification and the potential for ammonium hydrogen sulfate formation. Many scholars have conducted research on flue gas denitrification using H_2_O_2_. Limvoranusorn et al.^6^ found that spraying an H_2_O_2_ solution directly into the flue gas can achieve a high NO oxidation rate. Wang et al.^7^ leveraged metal surfaces to enhance the thermal decomposition of hydrogen peroxide and the oxidation of NO. Results indicated that the evaporation rate and decomposition rate of hydrogen peroxide significantly influence NO oxidation. Kou et al.^8^ activated H_2_O_2_ vapor thermally to generate highly oxidative hydroxyl radicals (OH) and introduced them into the flue gas. They concluded that thermal activation of hydrogen peroxide is feasible for NO oxidation, while the temperature and flow rate of the nitrogen carrier gas have a notable impact on the conversion efficiency. Li et al.^9^ established a model for the oxidation of NO and SO_2_ in coal-fired flue gas by hydrogen peroxide. They investigated the effects of temperature and hydrogen peroxide concentration on the oxidation of NO and SO_2_, determining that the optimal temperature ranges for NO and SO_2_ oxidation are 650–920 K and 650–750 K, respectively. In the current research landscape, it has been observed that the optimal catalytic temperature for the oxidation removal of NO through H_2_O_2_ is considerably high, which is inadequate to fulfill the denitrification demands during deep load-following periods of power units.

Nevertheless, recent studies have highlighted that the implementation of specific catalytic strategies can facilitate the decomposition of H_2_O_2_ into hydroxyl free radicals (OH) under low-temperature conditions, thereby enhancing the denitrification efficacy of H_2_O_2_. Hao et al.^10^ leveraged ultraviolet radiation to facilitate the decomposition of H_2_O_2_ into hydroxyl free radicals, discovering that under optimal conditions, a desulfurization efficiency of 100% and a denitrification efficiency of 87.8% could be achieved. However, the equipment and operational costs associated with catalyzing the decomposition of H_2_O_2_ using ultraviolet radiation are excessively high. On the other hand, Wang et al.^11^ conducted denitrification experiments using a Cu^2+^/Fe^2+^-H_2_O_2_ catalytic system. The experimental results indicated that Fe^2+^ and Cu^2+^ can promote the decomposition of H_2_O_2_ enhancing the removal efficiency of NO. The utilization of metal ions to prepare catalysts for the catalytic decomposition of H_2_O_2_ presents a lower cost compared to the ultraviolet irradiation method, making it a more worthy candidate for industrial promotion.

The study establishes a 350 kW thermal state experimental system, employing low-temperature hydrogen peroxide (H_2_O_2_) catalytic oxidation coupled with wet flue gas desulfurization technology. This methodology aligns closely with the operational conditions of peak regulation units and enables the integrated removal of NO_x_ and SO_x_ using a unified pollutant catalytic oxidation system. The research investigates the comprehensive mechanisms of pollutant removal, aiming to enhance pollution control in low-load operational units significantly.

## Experimental system and method

### Experiment system

**Figure 1:**
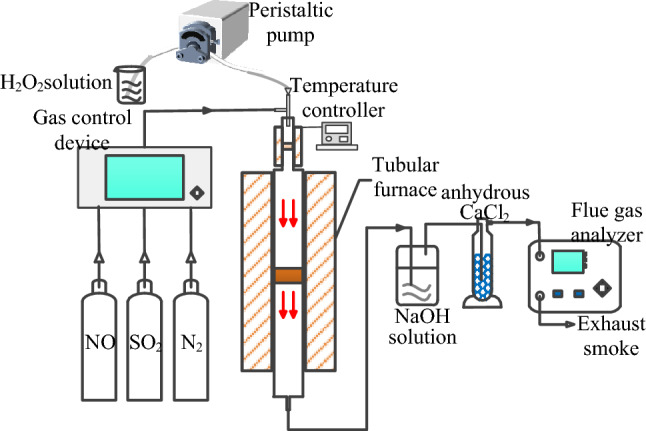
Schematic diagram of simulated flue gas experimental system.

As depicted in Fig. 1, the simulated flue gas experimental system is primarily designed for catalyst screening under laboratory conditions. This experimental setup comprises a gas distribution system, a vertical tube furnace, a quartz glass reactor, an H_2_O_2_ gasification system, an exhaust gas treatment device, and a flue gas analyzer. The fundamental procedure of the experiment involves pre-fabricating simulated flue gas composition through the gas distribution system. The gas components are thoroughly mixed using a gas control device. Subsequently, an H_2_O_2_ solution is pumped into the gasification device by a peristaltic pump. In the quartz reactor, the gasified mixture undergoes catalytic oxidation, facilitated by the catalyst, which converts pollutants such as NO into other forms, including NO_2_. This process is a crucial step in altering the chemical composition of the pollutants, leading to their subsequent removal or conversion into less harmful substances. Finally, the treated flue gas is absorbed by NaOH solution and dried with anhydrous calcium chloride before being directed into the flue gas analyzer for analysis.

**Figure 2:**
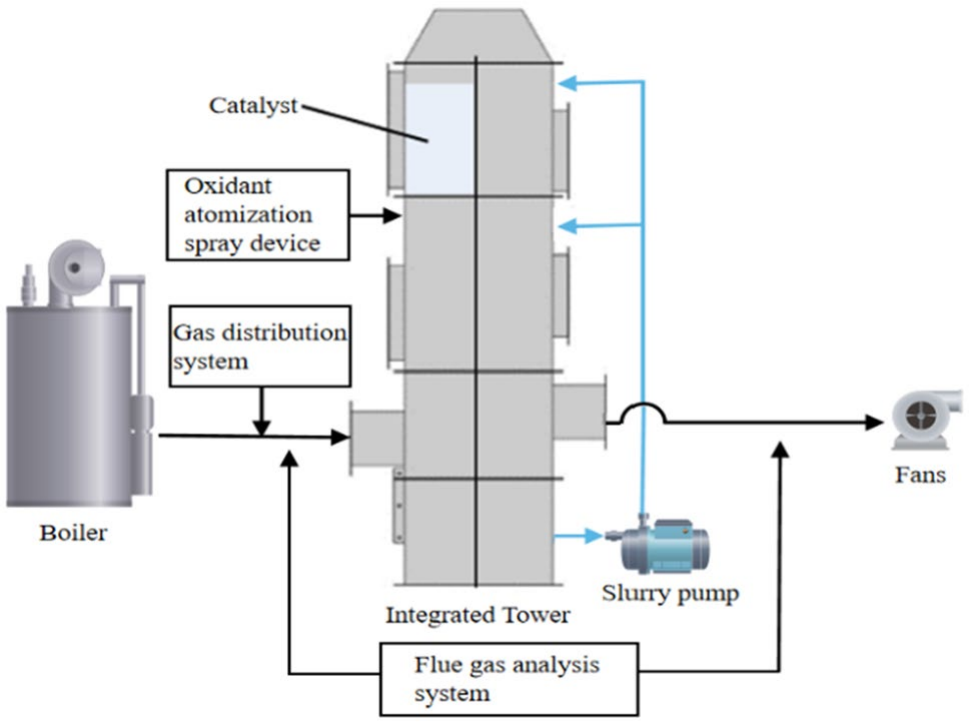
Integrated experimental system for removal of industrial pollutants.

This study has developed an industrial-scale integrated pollutant removal experimental system, as illustrated in Fig. 2. The experimental setup comprises a 350 kW hot water boiler, a gas distribution system, an integrated tower, an oxidant atomizing spray device, a flue gas analysis system, a slurry pump, and a fan. The fundamental experimental procedure is as follows: high-temperature flue gas is generated by the hot water boiler and controlled to the desired temperature using a heat exchanger before entering the integrated tower. Inside the integrated tower, the flue gas is mixed with the atomized oxidant, ensuring thorough counter-flow mixing. The catalysts arranged within the tower catalytically oxidize pollutants, particularly nitrogen oxides, converting them into high-valence compounds. Subsequently, the absorbent slurry is introduced into the tower through spray pipes via a slurry pump, achieving extensive mixing with the oxidized flue gas and enabling efficient washing and removal of pollutants. The flue gas analysis system is employed to measure the composition and concentration of pollutants in the post-purification flue gas. Ultimately, the purified flue gas is discharged using a fan.

### Data processing method

1. NO oxidation efficiency
  1 $$ \eta_{{{\text{NO}}}} = \frac{{{\text{NO}}_{{{\text{in}}}} - {\text{NO}}_{{{\text{out}}}} }}{{{\text{NO}}_{{{\text{in}}}} }} \times 100\% $$
  where No_in_ is the inlet NO concentration, mg m^−3^; NO_out_ is the outlet NO concentration, mg m^−3^; *η*_NO_ is the NO oxidation efficiency, %.
2. NO_*x*_ removal efficiency
  2 $$ \eta_{{{\text{NO}}_{x} }} = \frac{{{\text{NO}}_{{{\text{in}}}} - {\text{NO}}_{{{\text{out}}}} - {\text{NO}}_{{2,{\text{out}}}} }}{{{\text{NO}}_{{{\text{in}}}} }} \times 100\% $$
  In the given equations: “NO_in_”—inlet NO concentration, mg m^−3^; NO_out_—outlet NO concentration, mg m^−3^; NO_2,out_—outlet NO concentration, mg m^−3^; η_NO*x*_—NO_*x*_ oxidation efficiency, %.
3. SO_2_ removal efficiency
  3 $$ \eta_{{{\text{SO}}_{{2}} }} = \frac{{{\text{SO}}_{{\text{2,in}}} - {\text{SO}}_{{\text{2,out}}} }}{{{\text{SO}}_{{\text{2,in}}} }} \times 100\% $$
  In the given equations: “SO_2in_”—inlet SO_2_ concentration, mg m^−3^; SO_2out_—outlet SO_2_ concentration, mg m^−3^; η_SO2_—SO_2_ oxidation efficiency, %.

### Catalyst preparation and characterization methods

Due to factors such as the tetravalent state of titanium in TiO_2_ and the stability of Ti–O bonds, the enhancement of TiO_2_'s catalytic performance in reactions is inherently limited. This limitation often leads to TiO_2_ being selected as a catalyst carrier. However, doping TiO_2_ with other metals can alter its lattice structure and introduce active sites for catalysis. Iron, as a doping metal, demonstrates an effective ability to substitute Ti, creating active sites on the TiO_2_ surface that promote the decomposition of H_2_O_2_ into ·OH radicals. The catalyst used in this experiment was prepared using the co-precipitation method to produce iron-loaded titanium dioxide, which was subsequently investigated for its catalytic oxidation denitrification performance at different loading ratios. The preparation procedure was as follows: firstly, nine-hydrate ferric nitrate (Fe(NO_3_)_3_·9H_2_O, China National Pharmaceutical Chemical Reagents Co., Ltd.) and titanium sulfate powder (Ti(SO_4_)_2_, China National Pharmaceutical Chemical Reagents Co., Ltd.) were precisely weighed according to the required Fe/Ti molar ratio using an electronic balance. A specific quantity of deionized water at 0 °C was used to dissolve and homogeneously mix the two powders. The mixture was continuously stirred at a constant temperature in a water bath for 1 h. Subsequently, 25 wt% ammonia solution was gradually added dropwise while stirring until the pH of the solution reached 10. The stirring process was continued for another 1 h to ensure complete precipitation. The resulting precipitate was then subjected to thorough washing with deionized water using a vacuum filtration device until the pH of the washout solution was in the range of 7 to 8. Afterward, the washed precipitate was dried in a 105 °C drying oven for 12 h to achieve complete dehydration. Upon cooling, the dried material was preliminarily ground into a powdered form, which was subsequently placed in a high-temperature muffle furnace (JZ-4-1200, Shanghai Jingzhao Machinery Equipment Co., Ltd., China) for calcination at 400 °C for 6 h to enhance the catalyst's activity. Finally, the calcined sample was finely ground and sieved to obtain the catalyst sample with the desired loading ratio. Catalyst samples were prepared with Fe/Ti molar ratios of 0%, 0.5%, 1%, 2%, and 3% using the aforementioned method.

**Figure 3:**
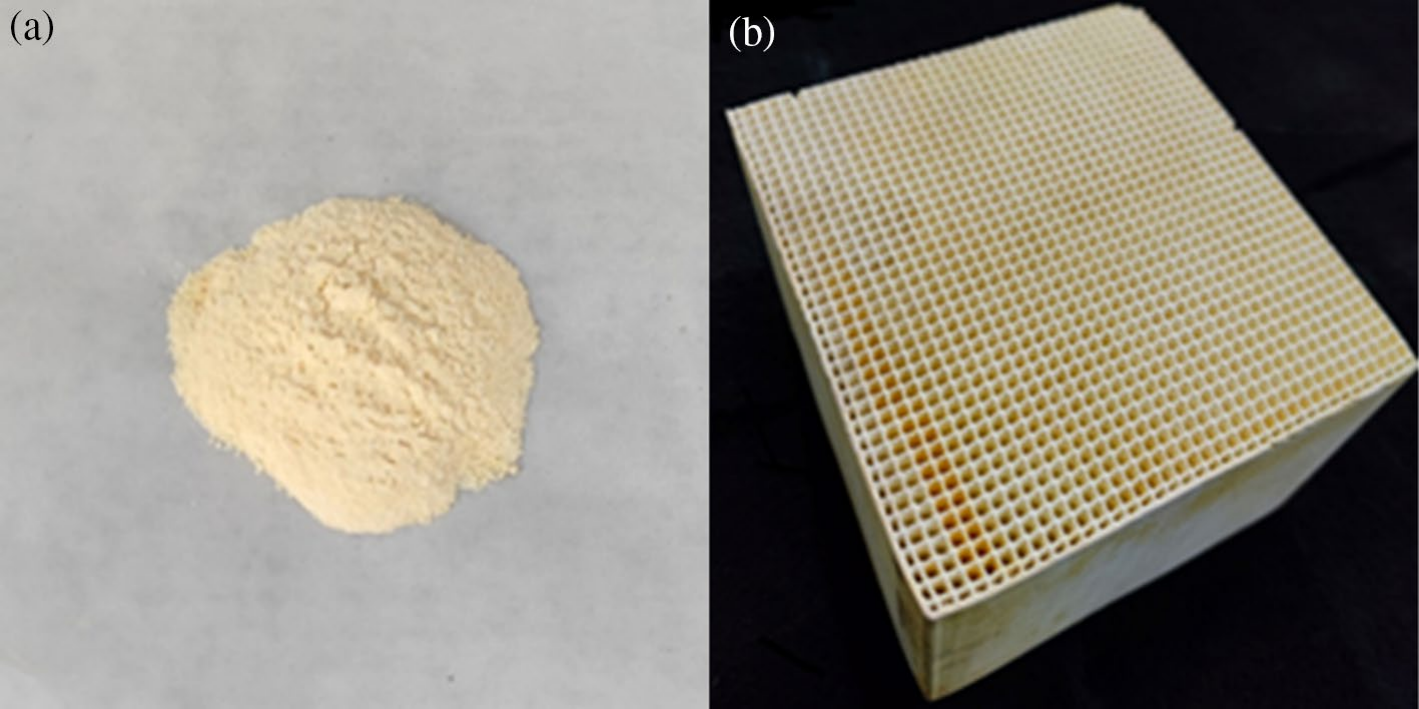
(**a**) Fe/TiO_2_ powder sample. (**b**) Fe/TiO_2_ honeycomb skeleton finished product.

After screening the catalysts prepared using the co-precipitation method through the simulated flue gas experimental system, a catalyst formulation with outstanding performance was identified. Based on this formulation, a honeycomb-like cordierite (Ruilan Environmental Technology Co., Ltd., China) structure with a pore size of 3Fmm (100-200CPSI), commonly used in industrial catalysis, was selected as the carrier. The preparation involved a coating process, in which the Fe/TiO_2_ catalyst powder was loaded onto the honeycomb-like cordierite carrier. The Fe/TiO_2_ catalyst powder is shown in Fig. 3a, and the honeycomb catalyst supported by Fe/TiO_2_ is shown in Fig. 3b.

In this study, the catalyst's material composition and internal atomic or molecular structure were investigated using an XRD-6100 X-ray diffractometer manufactured by Shimadzu Corporation, Japan. The catalyst's morphology and microscale dimensions were examined with an SU8230 scanning electron microscope produced by Hitachi, Japan. Furthermore, the catalyst's specific surface area, pore size, pore size distribution, and nitrogen adsorption–desorption isotherms before and after the reaction were analyzed using a JW-TB440 instrument, designed for characterizing surface properties and pore structures of micro-nano materials, provided by Beijing Jingwei Gaobo Technology Co., Ltd. These advanced instruments were employed to comprehensively explore the microstructural changes of the catalyst before and after the reaction.

## Results and discussion

Under the low-load operation conditions of the deep load-following power generation unit, the reduction in boiler fuel quantity leads to a decrease in the furnace outlet temperature. Without appropriate measures, the conventional Selective Catalytic Reduction (SCR) denitrification efficiency would be significantly reduced, and the SCR system might even fail to operate properly. Hydrogen Peroxide (H_2_O_2_) oxidation denitrification can effectively overcome this drawback and can serve as one of the alternative technologies for pollutant removal under low-load conditions. To further investigate the integrated pollutant removal performance of H_2_O_2_ in the presence of Fe/TiO_2_ catalyst, an experimental system for integrated pollutant removal was established using an integrated tower. This study explores the influence of flue gas temperature, catalyst space velocity, H_2_O_2_/(SO_2_ + NO) molar ratio, initial concentrations of NO and SO_2_, H_2_O content, liquid-to-gas ratio, and absorption solution pH on NO oxidation efficiency, NO_x_ removal efficiency, and SO_2_ removal efficiency.

### Catalyst screening experiment

**Figure 4:**
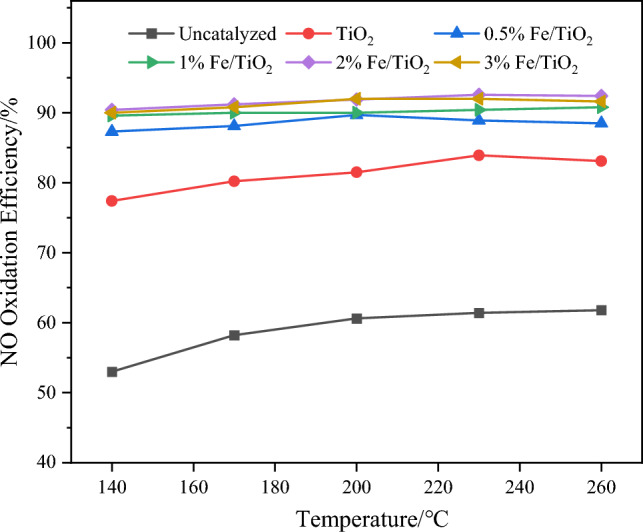
Effect of different Fe loading ratios on NO oxidation efficiency.

**Figure 5:**
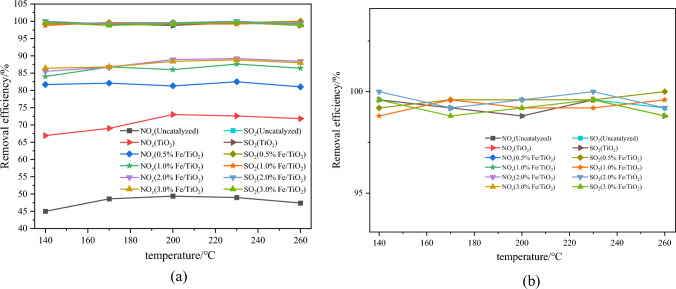
Effect of different Fe loading ratios on pollutant removal efficiency (**a**) and magnified section image (**b**).

The experimental conditions involved a total flue gas flow rate of 1000 mL min^−1^, a space velocity of 30000 h^−1^, H_2_O_2_ gasification temperature of 140 °C, 10 wt% H_2_O_2_, and an H_2_O_2_/(SO_2_ + NO) molar ratio of 4. The initial concentration of NO was 335 mg m^−3^, the initial concentration of SO_2_ was 714 mg m^−3^, and N_2_ was used as the balance gas. The absorbent solution used was 600 mL of 0.2 mol L^−1^ NaOH. Figures 4 and 5 illustrate the variations in NO oxidation efficiency, NO_*x*_ removal efficiency, and SO_2_ removal efficiency with increasing temperature for different loading ratios.

From Fig. 4, it is evident that the addition of catalyst markedly enhances the NO oxidation efficiency. Taking the temperature of 200 °C as an example, compared to the scenario without catalyst, the inclusion of TiO_2_ catalyst results in an increase in NO oxidation efficiency from 60.6 to 81.5%. Moreover, the efficiency is further augmented with the incorporation of catalyst loaded with Fe, reaching approximately 92% at a loading ratio of 3%.

From Fig. 5, it is evident that, at the same temperature, the NO_*x*_ removal efficiency does not exhibit a significant improvement with increasing Fe loading ratio. The underlying reason for this can be attributed to the fact that the loading of Fe ions can indeed stimulate more oxygen vacancies, thereby enhancing the catalytic activity of the catalyst. However, an excessive loading of Fe may lead to pore blockage in the catalyst, resulting in a decrease in catalytic performance. As for the removal of SO_2_, it is almost completely removed both with and without the catalyst, indicating that NaOH is highly effective in absorbing SO_2_. Even when considering the maximum instrument error, the SO_2_ removal efficiency exceeds 98%. At a loading ratio of 2% Fe, the NO oxidation efficiency ranges from 90.4 to 92.6%, and the NO_*x*_ removal efficiency varies between 85.5 and 90%. With the increase in reaction temperature, there is a slight decrease in both NO oxidation and removal efficiency. This can be mainly attributed to the increased ineffective decomposition of H_2_O_2_ in the system at higher temperatures, which, to some extent, reduces the catalytic oxidation effect and subsequently leads to a decline in removal efficiency. However, within the temperature range of 140 to 260 °C, the changes in the catalytic oxidation and removal efficiency are relatively minor, indicating the catalyst's capability for pollutant removal under low-temperature conditions. Based on these observations, the Fe/TiO_2_ catalyst with a loading ratio of 2% Fe is selected as the catalyst type for subsequent experiments in this study.

### Effect of temperature on pollutant removal efficiency

**Figure 6:**
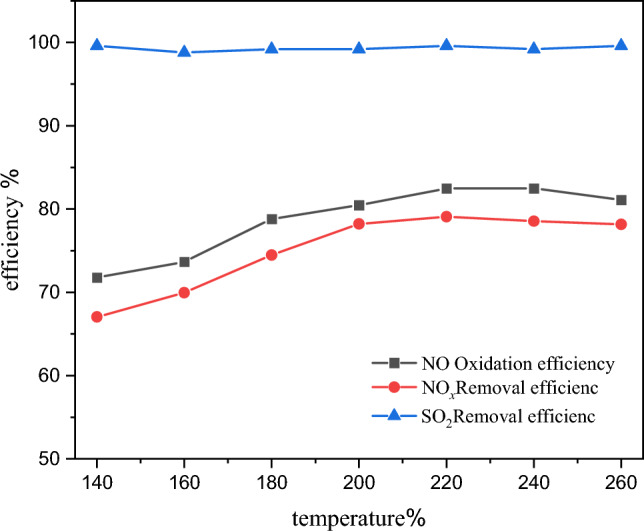
Effect of temperature on NO oxidation and pollutant removal efficiency.

The flue gas conditions were maintained consistent with the actual low-load operation conditions, except for particulate matter and flow rate. The remaining experimental conditions are consistent with those detailed in "Catalyst screening experiment" section. The variations of NO oxidation efficiency, NO_*x*_ removal efficiency, and SO_2_ removal efficiency with temperature are depicted in Fig. 6.

It is evident that as the flue gas temperature increases, almost complete removal of SO_2_ is achieved, indicating that the variation in temperature due to changes in the inlet conditions has negligible impact on SO_2_ removal efficiency in the catalytic section. Conversely, both NO oxidation efficiency and NO_*x*_ removal efficiency exhibit an increasing trend with rising temperature. For instance, when the flue gas temperature rises from 140 to 200 °C, the NO oxidation efficiency increases from 71.8 to 80.5%, while the NO_*x*_ removal efficiency increases from 67 to 78.2%. The primary reasons for these enhancements are as follows: first, the degree of H_2_O_2_ gasification gradually improves with increasing temperature; second, the chemical reaction rate accelerates, leading to more generation of ·OH on the catalyst surface, which contributes to an enhanced oxidation efficiency of the system. However, the temperature increase also results in the ineffective decomposition of H_2_O_2_, generating O_2_ and H_2_O, thereby reducing the concentration of H_2_O_2_ in the system. In summary, beyond 200 °C, the NO oxidation efficiency and NO_x_ removal efficiency remain relatively stable, reaching approximately 82% and 79%, respectively.

### Effect of catalyst space velocity ratio on pollutant removal efficiency

**Figure 7:**
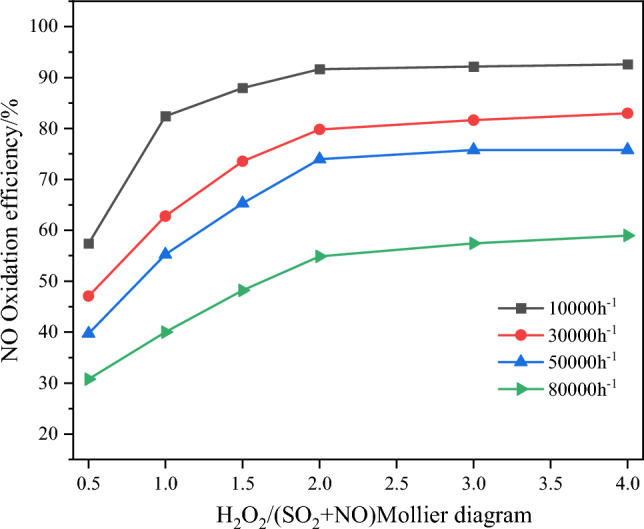
Effect of catalyst space velocity ratio on NO oxidation efficiency.

**Figure 8:**
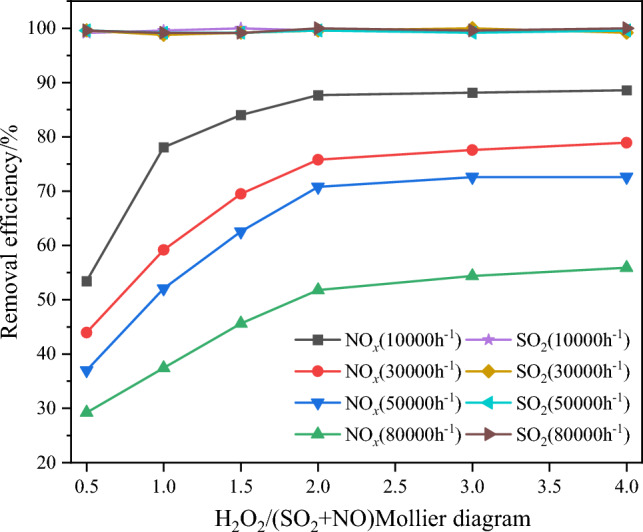
Effect of catalyst space velocity ratio on pollutant removal efficiency.

The flue gas temperature was set at 230 °C, with all other experimental conditions remaining consistent with those described in "Catalyst screening experiment" section. NO_x_ removal efficiency, and SO_2_ removal efficiency with different H_2_O_2_/(SO_2_ + NO) molar ratios at various space velocities are illustrated in Figs. 7 and 8.

From Fig. 8, it is evident that SO_2_ is almost completely removed. Both the space velocity and H_2_O_2_/(SO_2_ + NO) molar ratio significantly influence NO oxidation and removal efficiency. Under the same space velocity conditions, increasing the H_2_O_2_/(SO_2_ + NO) molar ratio from 0.5 to 2 results in a noticeable enhancement in NO oxidation efficiency and removal efficiency. This effect can be attributed to the fact that, at lower molar ratios, the system contains a lower amount of H_2_O_2_, thus increasing H_2_O_2_ leads to a more pronounced improvement in catalytic oxidation efficiency. However, when the molar ratio exceeds 2, the increase in NO oxidation and NO_*x*_ removal efficiency becomes gradual and tends to stabilize. Under the same molar ratio conditions, a smaller space velocity of the catalyst corresponds to higher NO oxidation and removal efficiency. This is due to the fact that a lower space velocity implies more active sites on the catalyst, which enhances the ability of H_2_O_2_ to decompose into ·OH. As a result, more NO and NO_2_ are oxidized into higher-valence oxides. Additionally, a smaller space velocity corresponds to a higher proportion of active components on the catalyst. The excess active sites facilitate the reduction of NO_2_ back to NO during the catalytic oxidation process, as represented by Eqs. (4) and (5). This somewhat attenuates the enhancement in oxidation and removal efficiency resulting from reducing the space velocity. Considering the overall economic efficiency and NO catalytic oxidation removal efficiency, the subsequent experiments will utilize a catalyst with a space velocity of 10,000 h^−1^ and an H_2_O_2_/(SO_2_ + NO) molar ratio of 1.5. Under these conditions, the NO oxidation efficiency and NO_x_ removal efficiency are approximately 88% and 84%, respectively, while SO_2_ is almost completely removed.

4 $$ {\text{NO}}_{{2}} {\text{ + Ti}}^{{3 + }} \to {\text{Ti}}^{{4 + }} {\text{ + NO}} $$

5 $$ {\text{Ti}}^{{3 + }} { + } \cdot {\text{OH}} \to {\text{Ti}}^{{4 + }} {\text{ + OH}}^{ - } $$

### Effect of H_2_O_2_ on pollutant removal efficiency

**Figure 9:**
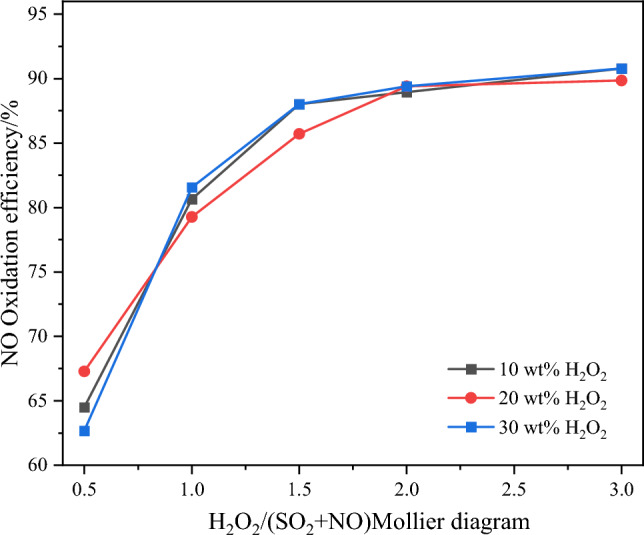
Effect of H_2_O on NO oxidation efficiency.

**Figure 10:**
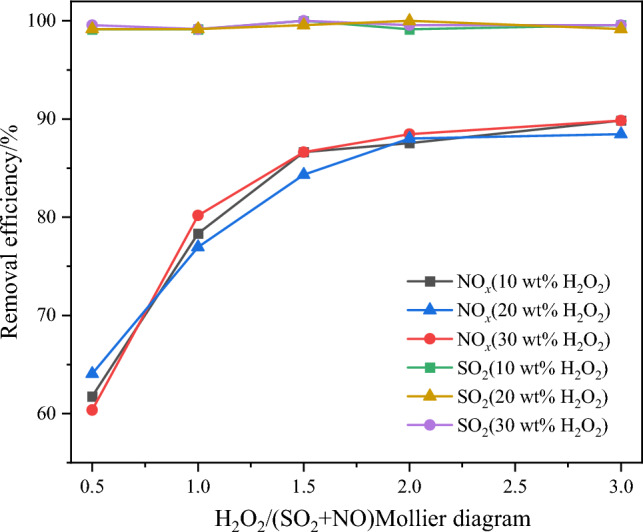
Effect of H_2_O on pollutant removal efficiency.

Under the conditions of the same H_2_O_2_/(SO_2_ + NO) molar ratio and initial concentrations of NO and SO_2_, H_2_O_2_ solutions with concentrations of 10 wt%, 20 wt%, and 30 wt% were utilized. Due to the variations in H_2_O_2_ concentrations, the flow rates were different, resulting in different amounts of H_2_O_2_ participating in the reaction. This investigation aims to explore the impact of H_2_O_2_ concentration on pollutant removal efficiency. The remaining experimental conditions were selected based on the optimal pollutant removal conditions identified in "Effect of catalyst space velocity ratio on pollutant removal efficiency" section. The results depicting the NO oxidation efficiency, NO_x_ removal efficiency, and SO_2_ removal efficiency under different H_2_O_2_ concentrations and H_2_O_2_/(SO_2_ + NO) molar ratios are presented in Figs. 9 and 10.

It is evident from the results that the SO_2_ removal efficiency is hardly affected by the water content in the system. The differences in NO oxidation and NO_x_ removal efficiency under different water content levels show only minor fluctuations within a narrow range, indicating no significant deviations in efficiency. Generally, the influence of water (H_2_O) on the catalytic oxidation effect of H_2_O_2_ mainly manifests in the competition between H_2_O and H_2_O_2_ for some active sites on the catalyst surface, leading to a reduction in the utilization efficiency of H_2_O_2_. Additionally, water vapor can diffuse into the catalyst pores and condense there through capillary action, further hindering the contact of H_2_O_2_ on the catalyst surface. However, based on the results of this experiment, it can be concluded that the water content has not exerted a significant impact on the pollutant removal efficiency, indicating that the selected catalyst in this study possesses certain water tolerance. Consequently, the subsequent experiments will continue using the 10 wt% H_2_O_2_ solution to further investigate the pollutant removal behavior.

With the same H_2_O_2_ concentration, a significant increase in both NO oxidation efficiency and NO_*x*_ removal efficiency is observed as the H_2_O_2_/(SO_2_ + NO) molar ratio increases from 0.5 to 1.5. Taking 10 wt% H_2_O_2_ as an example, the NO oxidation efficiency and NO_x_ removal efficiency increase from 64.5 and 61.7% to 88 and 86.6%, respectively. When the molar ratio exceeds 1.5, the efficiencies remain relatively constant. This behavior can be attributed to the gradual excess of H_2_O_2_ with an increase in the H_2_O_2_/(SO_2_ + NO) molar ratio, which leads to the occurrence of self-consumption reactions among ·OH, ·OOH, and H_2_O_2_, as described in Eqs. (6) to (9) below. This phenomenon results in a decreased utilization efficiency of ·OH, thereby limiting further enhancement of NO catalytic oxidation efficiency with an increasing dosage of H_2_O_2_.

6 $$ {\text{OH + }} \cdot {\text{OH}} \to {\text{H}}_{{2}} {\text{O}}_{{2}} $$

7 $$ {\text{OH + H}}_{{2}} {\text{O}}_{{2}} \to {\text{H}}_{{2}} {\text{O + }} \cdot {\text{OOH}} $$

8 $$ {\text{OH + }} \cdot {\text{OOH}} \to {\text{H}}_{{2}} {\text{O + O}}_{{2}} $$

9 $$ {\text{2H}}_{{2}} {\text{O}}_{{2}} \to {\text{2H}}_{{2}} {\text{O + O}}_{{2}} $$

### Effect of SO_2_ initial concentration on pollutant removal efficiency

**Figure 11:**
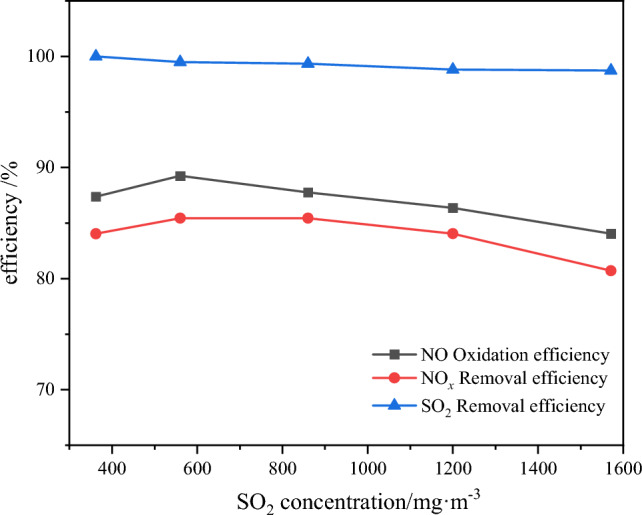
Effect of SO_2_ on NO oxidation and pollutant removal efficiency.

Under typical conditions, when the load of the peak load regulation unit is below 30%, the boiler outlet SO_2_ concentration usually falls within the range of 500 to 1000 mg m^−3^. Hence, the selected initial SO_2_ concentration range is set from 360 to 1570 mg m^−3^. The remaining experimental conditions were chosen based on the optimal conditions established earlier in the text. the variations of NO oxidation efficiency and pollutant removal efficiency with respect to the SO_2_ concentration are depicted in Fig. 11.

10 $$ {\text{2SO}}_{{2}} + {\text{O}}_{{2}} \to {\text{2SO}}_{{3}} $$

11 $$ {\text{SO}}_{{3}} + {\text{H}}_{{2}} {\text{O}} \to {\text{H}}_{{2}} {\text{SO}}_{{4}} $$

12 $$ {\text{NO}}_{{2}} + {\text{SO}}_{{2}} \to {\text{SO}}_{{3}} + {\text{NO}} $$

13 $$ {\text{NO}}_{{2}} + {\text{SO}}_{{3}} \to {\text{NO}} + {\text{SO}}_{{4}}{^{{{2}} - }} $$

14 $$ \cdot {\text{OH}} + {\text{NO}} \to {\text{HNO}}_{{2}} $$

15 $$ \cdot {\text{OH}} + {\text{NO}}_{{2}} \to {\text{HNO}}_{{3}} $$

The results demonstrate that as the initial SO_2_ concentration increases from 360 to 560 mg m^−3^, both the NO oxidation efficiency and NO_x_ removal efficiency exhibit an increment from 87.4 and 84% to 89.2 and 85.4%, respectively, after which they tend to stabilize^12^. However, when the SO_2_ concentration exceeds 860 mg m^−3^, the NO_*x*_ removal efficiency gradually decreases, reaching 80.7% at an SO_2_ concentration of 1570 mg m^−3^, while the NO oxidation efficiency remains relatively constant at 84%. At lower SO_2_ concentrations, the promotion of NO oxidation is attributed to the partial reduction of Fe^3+^ on the catalyst surface to Fe^2+^ by SO_2_, creating active sites that enhance the generation of ·OH and subsequently increase both the NO oxidation efficiency and NOx removal efficiency^13^. Conversely, there is a competition between SO_2_ and NO for H_2_O_2_ utilization. As the SO_2_ concentration gradually increases, it competes with NO for H_2_O_2_, leading to a depletion of available H_2_O_2_. Additionally, the presence of SO_2_ can partially block the oxygen vacancies on the catalyst surface, thereby reducing the efficiency of NO oxidation and removal at higher SO_2_ concentrations. It is noteworthy that when the H_2_O_2_ content in the system remains constant, the SO_2_ removal efficiency decreases beyond an initial SO_2_ concentration of 860 mg m^−3^^14^.

### Effect of initial concentration of NO on pollutant removal efficiency

**Figure 12:**
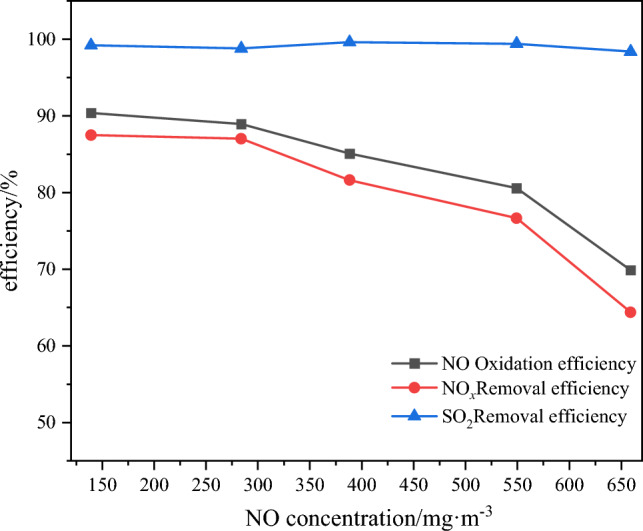
Effect of NO concentration on oxidation efficiency and pollutant removal efficiency.

Typically, for in-depth peak load regulating units, the NO concentration at the boiler outlet ranges from 300 to 600 mg m^−3^ when operating at a load below 30%. Therefore, in this experiment, the initial NO concentration was selected within the range of 140 to 650 mg m^−3^. The remaining experimental conditions were similarly selected based on the optimal conditions derived from the previous sections. The variations of NO oxidation efficiency and pollutant removal efficiency with respect to the NO concentration are depicted in Fig. 12.

### Effect of liquid–gas ratio on pollutant removal efficiency

**Figure 13:**
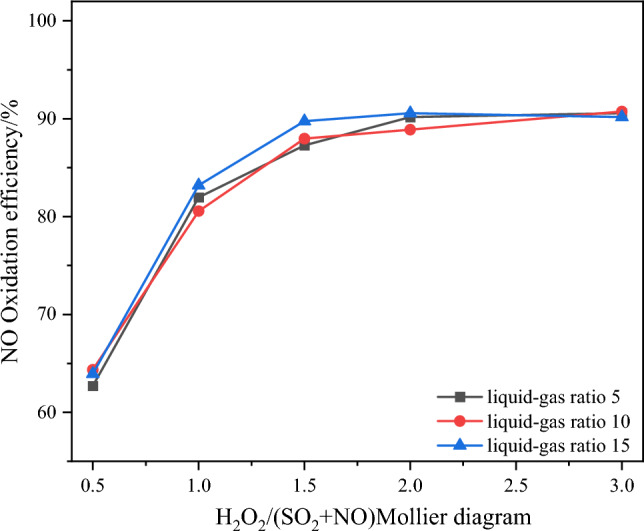
Effect of liquid–gas ratio on NO oxidation efficiency.

**Figure 14:**
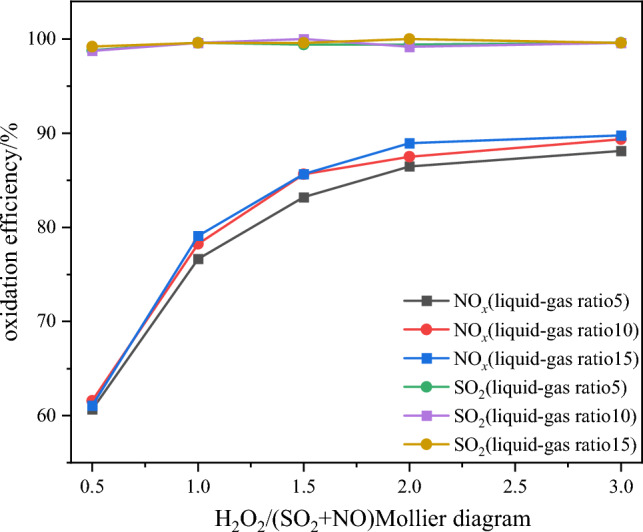
Effect of liquid–gas ratio on pollutant removal efficiency.

Keeping all other experimental conditions constant, the experiments were conducted by adjusting the liquid-to-gas ratio. the variations of NO oxidation efficiency, NO_x_ removal efficiency, and SO_2_ removal efficiency with different liquid-to-gas ratios are presented in Figs. 13 and 14^15,16^.

From Fig. 13, it can be observed that the NO oxidation efficiency remains relatively constant under different liquid-to-gas ratio conditions. This behavior is attributed to the direct influence of the liquid-to-gas ratio on the washing water quantity of the integrated tower's absorption side, which subsequently affects the removal efficiency of NO_x_ and SO_2_. On the other hand, Fig. 14 demonstrates that the SO_2_ removal efficiency is not significantly affected by the liquid-to-gas ratio. When the H_2_O_2_/(SO_2_ + NO) molar ratio increases from 0.5 to 1, the three different liquid-to-gas ratios exhibit relatively consistent removal efficiencies. However, with further increases in the H_2_O_2_/(SO_2_ + NO) molar ratio, beyond 1.5, the liquid-to-gas ratios of 10 and 15 demonstrate higher NO_*x*_ removal efficiencies compared to the liquid-to-gas ratio of 5. The underlying reason for this trend lies in the depth of the catalytic oxidation reaction. A larger liquid-to-gas ratio corresponds to a higher amount of absorption solution sprayed per unit time, leading to a more pronounced washing and removal effect. Ultimately, the NO_*x*_ removal efficiency stabilizes at around 88%, and the SO_2_ is almost completely removed. Moreover, it is believed that due to the lower temperature on the absorption side, some components such as HNO_2_, HNO_3_, and H_2_SO_4_ will undergo condensation after oxidation, further contributing to the removal effect. After comprehensive analysis, a liquid-to-gas ratio of 10 is chosen, which corresponds to a molar ratio of 1.5, resulting in an NO_x_ removal efficiency of 85.6%^17,18^.

### The influence of the pH value of the absorption solution on the pollutant removal efficiency

**Figure 15:**
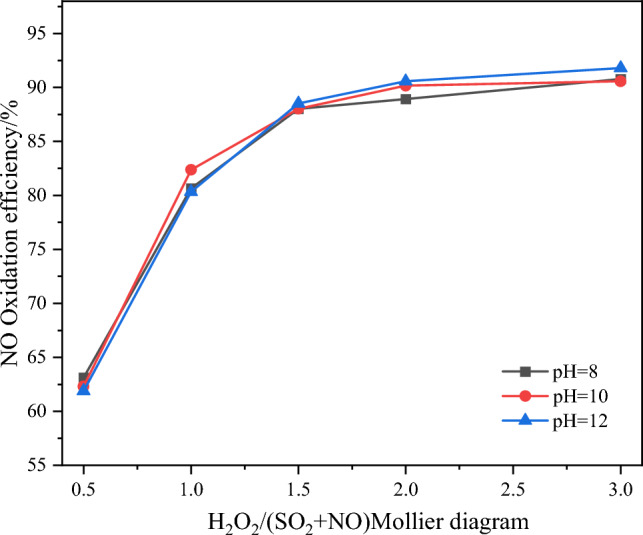
Effect of pH value of absorption solution on NO oxidation efficiency.

**Figure 16:**
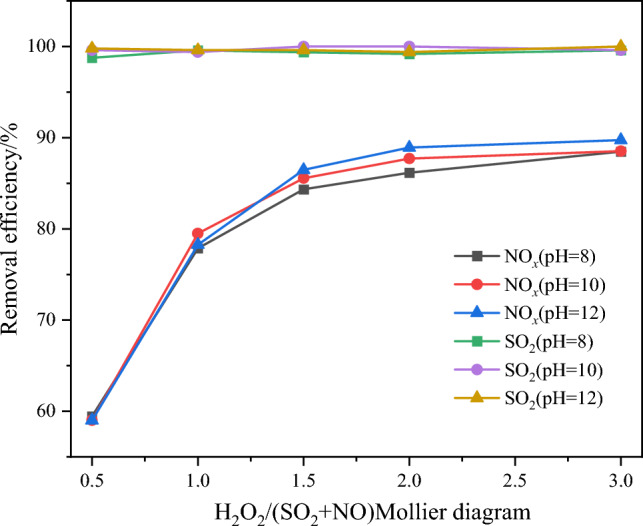
Effect of pH value of absorption solution on pollutant removal efficiency.

Experiments were conducted under the optimal conditions derived from the previous sections, The variations of NO oxidation efficiency, NO_*x*_ removal efficiency, and SO_2_ removal efficiency with different absorption solution pH values are presented in Figs. 15 and 16.

Figure 15 shows that under the same H_2_O_2_/(SO_2_ + NO) molar ratio conditions, the absorption solution pH has minimal impact on the NO oxidation efficiency. Instead, the absorption solution pH primarily influences the pollutant removal efficiency on the absorption side of the integrated tower. From Fig. 16, it can be observed that when the H_2_O_2_/(SO_2_ + NO) molar ratio is greater than 1.5, the absorption solution pH significantly affects the NO_*x*_ and SO_2_ removal efficiencie^.^ As the absorption solution pH increases, the corresponding NO_x_ removal efficiency under the same molar ratio conditions also increases. This can be attributed to the higher OH- concentration in the absorption solution at higher pH levels, which enhances the ability to absorb and remove acidic oxidants, such as HNO_2_, HNO_3_, and H_2_SO_4_, from the flue gas. Moreover, the spraying effect on the absorption side allows for the combination and condensation of some acidic oxidants in the flue gas with water vapor, as well as the condensation removal of HNO_2_, HNO_3_, and H_2_SO_4_. These processes contribute to the overall pollutant removal efficiency, leading to only minor changes in efficiency with varying pH levels. Considering the economic viability, stability, and continuity of the removal process, it is recommended to choose an absorption solution pH of 10. This pH value strikes a balance between effective pollutant removal and the cost-effectiveness of adding alkaline solution^19^.

### Catalyst performance testing and characterization

**Figure 17:**
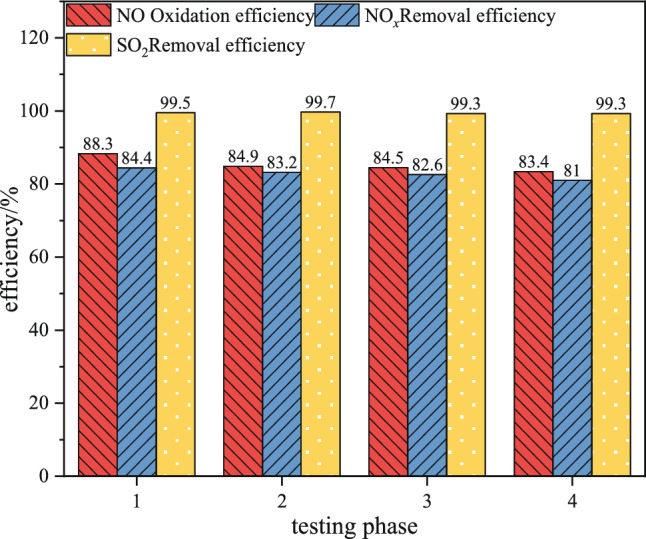
Oxidation and removal efficiency of pollutants in various performance test stages of catalysts.

To further explore the practical performance of the catalyst, it underwent four sequential performance tests during the experimental period, denoted as "Test 1," "Test 2," "Test 3," and "Test 4." Under the specified conditions of a flue gas flow rate of 200 m^3^ h^−1^, a flue gas temperature of 230 °C, an initial NO concentration of 335 mg m^−3^, an initial SO_2_ concentration of 714 mg m^−3^, an H_2_O_2_/(SO_2_ + NO) molar ratio of 1.5, an empty bed residence time of 10000 h^−1^, a liquid-to-gas ratio of 10, and an absorption solution pH of 10, the variations in pollutant removal efficiency with the number of catalyst testing cycles are presented in Fig. 17.

It is evident that as the catalyst usage time increases, both the NO oxidation efficiency and NO_*x*_ removal efficiency show a decreasing trend, while the SO_2_ removal efficiency exhibits relatively minor fluctuations. Notably, the NO oxidation efficiency decreased significantly from 88.2% in the second test to 84.9% compared to the first test. However, in the subsequent three tests, there was only a marginal variation, stabilizing at approximately 84%. On the other hand, the NO_*x*_ removal efficiency remained consistently above 80% throughout all four tests. In order to understand the underlying reasons for the observed changes in NO oxidation efficiency and NO_*x*_ removal efficiency, we conducted thorough microscopic characterization and analysis of the catalyst at each stage^20,21^.

#### SEM characterization

**Figure 18:**
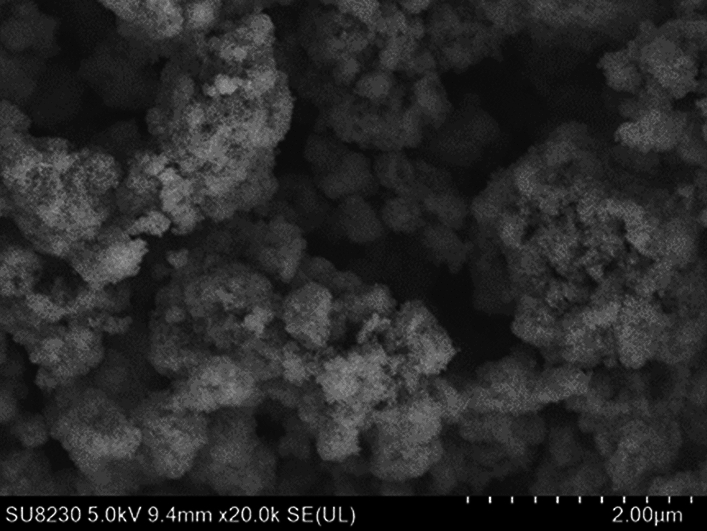
SEM image of fresh Fe/TiO_2_ catalyst.

**Figure 19:**
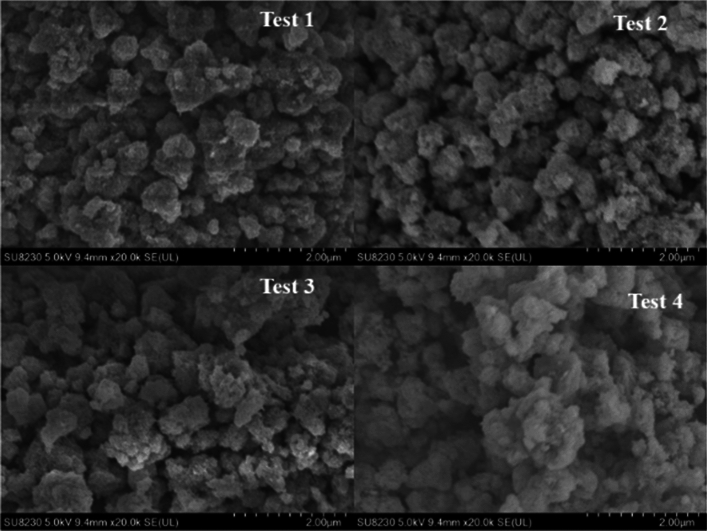
SEM images of catalysts in various performance testing stages.

To investigate the microscopic morphological changes of the Fe/TiO_2_ catalyst at various stages, we performed scanning electron microscopy (SEM) analysis on the fresh catalyst and samples taken at different testing stages after the reaction. The results are presented in Figs. 18 and 19.

From Fig. 18, it is evident that the structure of the fresh catalyst appears relatively porous, and the particles exhibit a relatively uniform distribution. Figure 19 reveals that, in comparison to the catalyst before the reaction, there is no significant alteration in the overall surface morphology. However, as the catalyst usage time increases, the particles become more tightly packed, and their volume undergoes expansion. This phenomenon is particularly pronounced in the SEM images of the catalyst after the fourth performance test. The primary reason behind this transformation is the occurrence of slight particle agglomeration during the catalyst's operational life. Specifically, during the reaction, SO_2_ on the catalyst surface reacts with H_2_O_2_ and·OH to form sulfate or sulfate salt precipitates. These precipitates continuously agglomerate and adsorb on the existing active surface, leading to partial blockage of some micropores and a reduction in catalytic efficiency^22^. To gain further insights into this phenomenon, X-ray fluorescence (XRF) analysis was conducted on the catalyst samples, with a specific focus on the sulfur (S) element content, as shown in Table 1. The results indicate a gradual increase in the S element content with the prolonged usage time of the catalyst.

**Table 1:** Sulfur element content of catalysts at each performance testing stage.

Sample	Fresh catalyst	Test 1	Test 2	Test 3	Test 4
Sulfur element content/wt%	2.27	2.40	3.03	3.06	3.06

Overall, the SEM and XRF analyses provide valuable insights into the changes in the catalyst's microstructure and elemental composition over multiple testing cycles, offering significant information on the reasons behind the variations in catalytic performance observed during the tests.

#### XRD characterization

**Figure 20:**
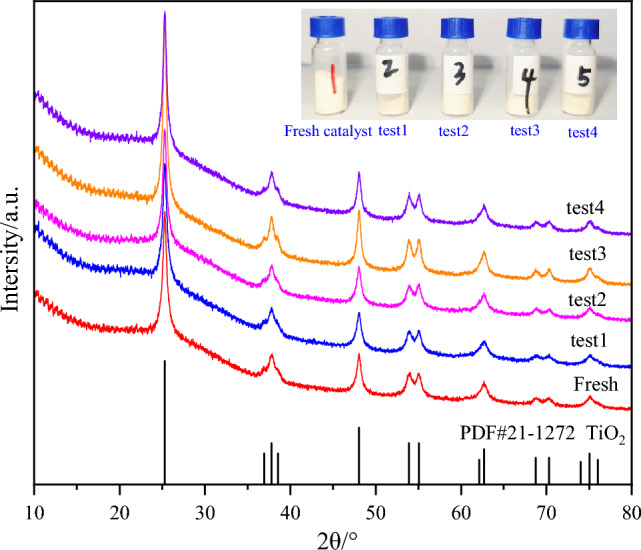
XRD patterns of catalysts in various performance test stages.

Figure 20 illustrates the X-ray diffraction (XRD) patterns of the Fe/TiO_2_ catalyst. It is evident that both the pre-reaction and post-reaction samples exhibit the main crystal phase corresponding to the rutile phase, as identified by the PDF standard card PDF#21-1272. No peaks corresponding to anatase or hematite structures are observed, and there are no prominent diffraction peaks of Fe ions, indicating that the loaded Fe did not alter the crystal phase of the catalyst and was successfully incorporated into the TiO_2_ structure. A comparison between the XRD patterns of the fresh catalyst and the catalyst after four performance tests reveals that the positions of the corresponding diffraction peaks remain unchanged, and no new diffraction peaks are observed. This finding suggests that the overall crystal structure of the catalyst remains largely unaffected after the reaction. The XRD results indicate that the Fe/TiO_2_ catalyst can maintain its original crystal structure after the reaction, thereby enhancing its resistance to deactivation^23,24^. Therefore, the XRD analysis demonstrates that the Fe/TiO_2_ catalyst retains its crystalline structure after the reaction, which contributes to its improved resistance against deactivation, ultimately enhancing its catalytic performance.

#### BET characterization

**Figure 21:**
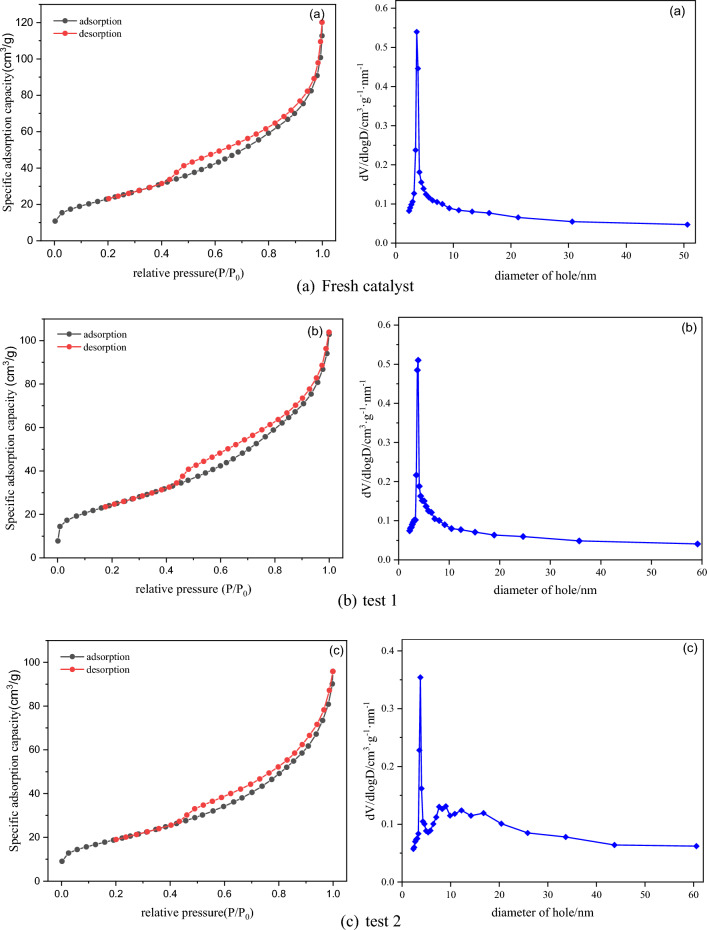

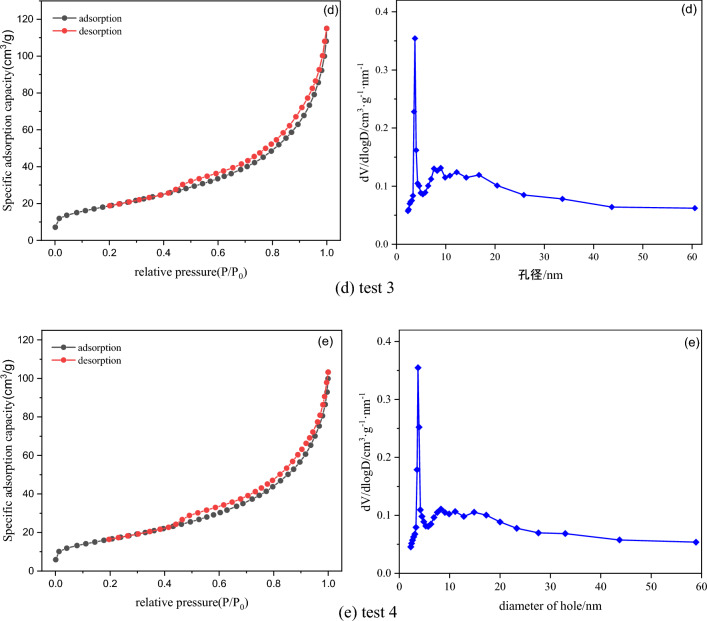
N_2_ adsorption–desorption curves and pore size distribution of catalysts at different performance tests.

Figure 21 depicts the N_2_ adsorption–desorption isotherms and BJH pore size distribution of the Fe/TiO_2_ catalyst before and after the reaction. A comparison of the isotherms obtained during the four performance tests reveals the occurrence of hysteresis loops within the range of relative pressure P/P_0_ = 0.43. According to the classification standards, the isotherms of the catalyst before and after the reaction belong to Type IV adsorption–desorption isotherms, with H3 hysteresis loops observed in both cases. The pore size distribution curves before and after the reaction show that the proportion of pores around 5 nm significantly decreases with increasing reaction time, while the number of pores with a size of around 10 nm increases. This phenomenon can be attributed to partial agglomeration occurring on the catalyst surface during the catalytic process, leading to the blockage of some mesopores, which is supported by the SEM and XRF results^25^. The specific surface area of the catalysts before and after the reaction, as indicated by BET analysis, is presented in Table 2. With increasing reaction time, the catalyst's specific surface area experiences a certain degree of reduction. Although the specific surface area alone cannot directly determine the catalyst's activity, a larger specific surface area promotes the generation of ·OH radicals, which can contribute to the catalytic process. Therefore, the decrease in specific surface area after the reaction is one of the factors contributing to the reduction in denitrification efficiency. In summary, the N_2_ adsorption–desorption isotherms and pore size distribution analysis reveal changes in the mesoporous structure of the Fe/TiO_2_ catalyst during the reaction, leading to reduced specific surface area and potentially affecting its catalytic performance^26,27^.

**Table 2:** BET specific surface area of catalysts in each performance testing stage.

Project	Specific surface area/m^2^ g^−1^
Fresh catalyst	84.84
Test 1	87.95
Test 2	69.04
Test 3	68.05
Test 4	60.11

## Conclusions

In this comprehensive study, we meticulously assessed the Fe-loaded TiO_2_ catalyst using laboratory-simulated flue gas conditions to determine optimal Fe loadings. The research entailed coating a honeycomb catalyst structure with Fe/TiO_2_ and examining its efficacy under low-load conditions in thermal power units, factoring in temperature and pollutant variables. The post-reaction catalyst was also structurally analyzed. Key findings include:

1. A 2% Fe loading on TiO_2_ demonstrated optimal results, achieving nearly full SO_2_ removal, with NO oxidation at 91.9% and NO_*x*_ removal at 88.9%, at a reaction temperature of 200 °C.
2. Between 180 and 260 °C, pollutant removal remained stable. With a constant space velocity, NO and NOx removal efficiency first increased with H_2_O_2_/(SO_2_ + NO) molar ratios, then stabilized. Notably, while low SO_2_ concentrations augmented NO oxidation, higher concentrations reduced NOx removal efficiency, competing with NO for H_2_O_2_. Optimal conditions were: H_2_O_2_/(SO_2_ + NO) molar ratio of 1.5, space velocity of 10,000 h^−1^, 10 wt% H_2_O_2_, liquid-to-gas ratio of 10, and pH of 10. Under these, SO_2_ removal was nearly complete with an 85.6% NO_*x*_ elimination rate.
3. Using the co-precipitation method, Fe ions were successfully incorporated into the TiO_2_ matrix. Remarkably, the crystalline structure of the catalyst remained largely unaltered before and after reactions. However, as the catalyst's usage duration extended, minor surface agglomeration was observed, leading to a slight reduction in pore diameter and specific surface area. Despite these alterations, throughout all testing phases, the NO oxidation efficiency consistently exceeded 83%, and the NO_*x*_ removal rate remained above 80%, underscoring the catalyst's robust oxidative stability.

## Data Availability

The datasets generated and analyzed during the current study are available from the corresponding author on reasonable request.
